# Rapid Responses and Mechanism of Action for Low-Dose Bisphenol S on *ex Vivo* Rat Hearts and Isolated Myocytes: Evidence of Female-Specific Proarrhythmic Effects

**DOI:** 10.1289/ehp.1408679

**Published:** 2015-02-26

**Authors:** Xiaoqian Gao, Jianyong Ma, Yamei Chen, Hong-Sheng Wang

**Affiliations:** Department of Pharmacology, University of Cincinnati College of Medicine, Cincinnati, Ohio, USA

## Abstract

**Background:**

Bisphenol S (BPS) has increasingly been used as a substitute for bisphenol A (BPA) in some “BPA-free” consumer goods and in thermal papers. Wide human exposure to BPS has been reported; however, the biological and potential toxic effects of BPS are poorly understood.

**Objective:**

In this study, we sought to elucidate the sex-specific rapid effect of BPS in rat hearts and its underlying mechanism.

**Methods:**

We examined the rapid effects of BPS in rat hearts using electrophysiology, confocal and conventional fluorescence imaging, and immunoblotting. Treatment was administered via acute perfusion of excised hearts or isolated cardiac myocytes.

**Results:**

In female rat hearts acutely exposed to 10^–9^ M BPS, the heart rate was increased; in the presence of catecholamine-induced stress, the frequency of ventricular arrhythmia events was markedly increased. BPS-exposed hearts showed increased incidence of arrhythmogenic-triggered activities in female ventricular myocytes and altered myocyte Ca^2+^ handling, particularly spontaneous Ca^2+^ release from the sarcoplasmic reticulum. The dose responses of BPS actions were inverted U-shaped. The impact of BPS on myocyte Ca^2+^ handling was mediated by estrogen receptor β signaling and by rapid increases in the phosphorylation of key Ca^2+^ handling proteins, including ryanodine receptor and phospholamban. The proarrhythmic effects of BPS were female specific; male rat hearts were not affected by BPS at the organ, myocyte, or protein levels.

**Conclusion:**

Rapid exposure to low-dose BPS showed proarrhythmic impact on female rat hearts; these effects at the organ, cellular, and molecular levels are remarkably similar to those reported for BPA. Evaluation of the bioactivity and safety of BPS and other BPA analogs is necessary before they are used as BPA alternatives in consumer products.

**Citation:**

Gao X, Ma J, Chen Y, Wang HS. 2015. Rapid responses and mechanism of action for low-dose bisphenol S on *ex vivo* rat hearts and isolated myocytes: evidence of female-specific proarrhythmic effects. Environ Health Perspect 123:571–578; http://dx.doi.org/10.1289/ehp.1408679

## Introduction

Bisphenols are a group of chemicals with two hydroxyphenyl functionalities in the structure (Liao and Kannan 2013). Bisphenols are widely used in the manufacturing industry, with bisphenol A (BPA) being the most commonly used member in the production of polycarbonate plastics and epoxy resins. Examples of BPA-based products include food containers, baby bottles, beverage and food can linings, thermal receipt paper, and water pipes. There is broad human exposure to BPA. Detectable levels of BPA have been found in urine and blood of > 90% of individuals examined in various sample populations (Vandenberg et al. 2007, 2012a; Ye et al. 2008).

BPA is an estrogenic endocrine-disrupting chemical (EDC). A large body of evidence has linked BPA exposure to human diseases such as cancer, diabetes, obesity, and various disorders in the reproductive, neuronal, immune, and cardiovascular (CV) systems (Diamanti-Kandarakis et al. 2009; Melzer et al. 2010; Zoeller et al. 2012). Because of the potential adverse health effects of BPA exposure, steps have been taken to reduce its use in consumer products in recent years. The European Union and the U.S. Food and Drug Administration have banned the use of BPA in baby bottles (European Commission 2011; Food and Drug Administration 2012), and France has issued a ban of manufacturing and sale of all food packaging containing BPA, starting in 2015 (Legifrance.gouv.fr 2012).

**Figure 1:**
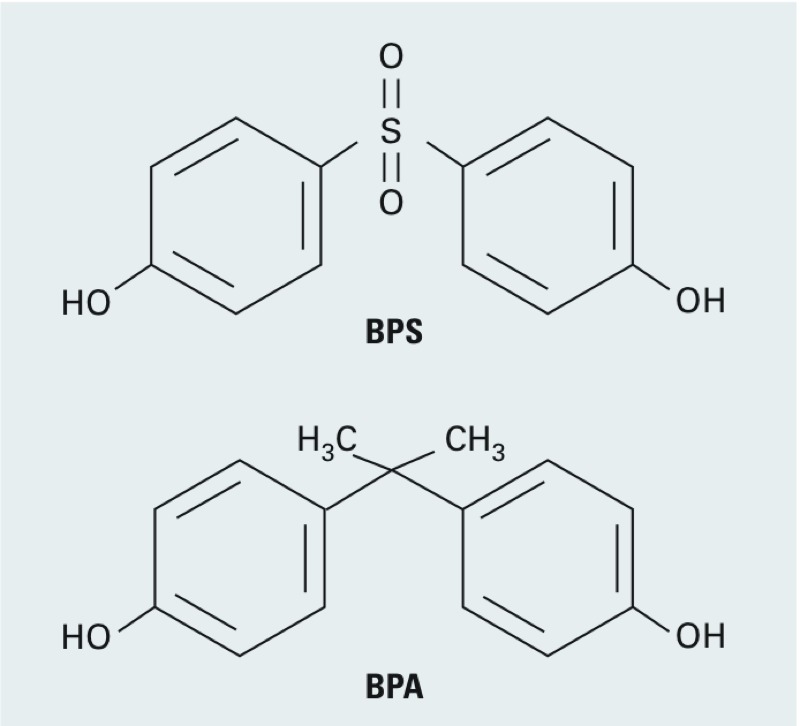
Structures of bisphenol S (BPS) and bisphenol A (BPA).

Bisphenol S (BPS; 4,4´-sulfonyldiphenol; CAS 80-09-1) is composed of two phenol groups on each side of a sulfonyl group, similar to BPA (Figure 1). BPS has increasingly been used as a substitute for BPA in some “BPA-free” consumer products. BPS is also found in thermal papers including receipts, envelopes, and airline boarding passes (Liao et al. 2012b). Although more heat stable and sunlight resistant than BPA, BPS still leaches from food cans and containers under normal use (Viñas et al. 2010). Human exposure to BPS has been reported and appears to be widespread. In a recent study of 315 urine samples collected in the United States and seven Asian countries from 2- to 84-year-old females and males, BPS was detected in 81% of the samples, with an overall mean urinary concentration of 0.65 ng/mL (2.6 nM) (Liao et al. 2012a). Similar to BPA, BPS has been found to have estrogenic activities (Grignard et al. 2012; Hashimoto et al. 2001). Although the health effects of BPA have been extensively studied, current knowledge on the potential biological effects and health impact of BPS is very limited.

Several epidemiological studies have shown associations between BPA exposure levels in adults and CV diseases or CV disease risk factors (Lang et al. 2008; Melzer et al. 2010, 2012). However, a lack of association between BPA exposure and CV disease was reported by LaKind et al. (2012). Previously, we reported that low-dose BPA rapidly promoted arrhythmogenic-triggered activities in ventricular myocytes from female rodents (Yan et al. 2011). Further, under stress or ischemic conditions, BPA increased the risk for cardiac arrhythmias in female rat hearts (Yan et al. 2011, 2013). These studies suggest a potential adverse impact of BPA on the CV system. The potential cardiac toxicity of BPS is entirely unknown. In the present study, we investigated the rapid impact of low-dose BPS on rodent hearts and cardiac myocytes, with a focus on the arrhythmogenic effect of BPS and its underlying cellular and molecular mechanisms.

## Materials and Methods

*Reagents*. We obtained BPS from Sigma-Aldrich. BPA (lot 111909; TCI America; ground by Battelle) was provided by the Division of the National Toxicology Program (National Institute of Environmental Health Sciences, National Institutes of Health). Methyl-piperidino-pyrazole {MPP; 1,3-bis(4-hydroxyphenyl)-4-methyl-5-[4-(2-piperidinylethoxy)phenol]-*1H*-pyrazole dihydrochloride; CAS 289726-02-9}, 2,3-bis(4-hydroxyphenyl)-propionitrile diarylpropionitrile (DPN; CAS 1428-67-7), and 4-[2-phenyl-5,7-bis(trifluoromethyl)pyrazolo[1,5-a]pyrimidin-3-yl]phenol (PHTPP; CAS 805239-56-9) were purchased from Tocris Bioscience. Other chemicals were obtained from Sigma-Aldrich unless otherwise stated. BPS and BPA were dissolved in DMSO as stock solutions (0.1 M) and stored at –20°C. Experimental solutions were prepared fresh using glass containers. All experimental apparatuses were free of polycarbonate plastic to avoid possible leaking of bisphenols into the solutions. We evaluated the effect of the highest concentration of DMSO vehicle used in the experiments (~ 10^–4^ M) and found no detectable effect on any of the end points we examined.

*Animals*. Adult Sprague-Dawley rats (200–250 g; Charles River) were used as nonsurviving sources of whole-heart preparations and isolated ventricular myocytes. Animals were housed two per cage in standard polycarbonate shoebox caging with Sani-chip bedding (Irradiated Aspen Sani-chip; P.J. Murphy Forest Products Corp.) to eliminate possible corn-based mycoestrogen exposure, and were fed *ad libitum* Teklad diet 2020 (Harlan Laboratories Inc.), which lacks soybean meal, alfalfa, or animal products that may introduce uncontrolled levels of estrogenic compounds. Animal room conditions included a 14-hr light:10-hr dark cycle (with lights on at 0600 hours and off at 2000 hours), 60% humidity, and a room temperature of 24°C. Newly arrived animals were housed in the animal facility for at least 2 weeks before surgery to allow for accommodation. Sterile drinking water was generated by a dedicated water purification system (Millipore Rios 16 with ELIX UV/Progard 2), which reduces oxidizable organics to < 1% of source levels. Glass bottles were used to dispense water. All animal procedures were performed in accordance with protocols approved by the University of Cincinnati Institutional Animal Care and Use Committee and followed recommendations of the Panel on Euthanasia of the American Veterinary Medical Association. Animals were treated humanely and with regard to alleviation of suffering.

A total of 75 animals was randomly allocated to experimental groups in this study, including 45 females and 30 males. These numbers, and those noted for individual experiments, do not include animals used in procedures or experiments that were unsuccessful. Experiments involving surface electrocardiography (ECG) measurements were performed throughout the regular business day (between 0900 and 1800 hours). For all other experiments, heart excisions were performed in the morning between 0900 and 1200 hours, and assessments of the cardiac myocytes were performed during the rest of the business day. All procedures were performed at approved satellite animal stations located in the laboratory. Animals used in this study were not previously used for other purposes. Treatment was administered to excised rat hearts and isolated ventricular myocytes via acute perfusion.

*Surface ECG*. Rats were anesthetized with sodium pentobarbital (80 mg/kg) by intraperitoneal injection. The hearts were rapidly excised, cannulated via the aorta, and perfused on a Langendorff apparatus with Krebs–Henseleit solution as previously described (Yan et al. 2011). The solution contained 118 mM NaCl, 4.7 mM KCl, 1.2 mM MgSO_4_, 1.2 mM KH_2_PO_4_, 0.5 mM EDTA, 2.5 mM CaCl_2_, 25 mM NaHCO_3_, and 11 mM glucose, pH 7.4, bubbled with 95% O_2_ and 5% CO_2_. Hearts were perfused at a pressure of 80 mmHg and a perfusion rate of approximately 15 mL/min. The surface ECG was continuously recorded from the heart, with one electrode positioned at the base and one at the apex of the heart. Data collection and analysis were performed using the Powlab 4/30 data acquisition system and LabChart 7 software (AD Instruments). Treatments were added to the perfusate after at least 1 hr of stabilization under control conditions; treatments included BPS, isoproterenol (Iso), BPS + Iso, or BPA + Iso for 30 min while the ECG was continuously recorded. Controls groups were treated with vehicle. The electrical rhythm of the heart was read from the recorded ECG, and the number of arrhythmic events within the treatment time, including ventricular tachycardia and premature ventricular beats, were counted. A total of 25 female hearts and 20 male hearts were used (total of 45 preparations in this experiment).

*Isolation of rat ventricular myocytes*. Ventricular myocytes were enzymatically dissociated from rat hearts using Langendorff perfusion as previously described (Yan et al. 2011). Briefly, the hearts were perfused with a Tyrode’s solution containing 0.7 mg/mL type II collagenase (Worthington Biochem). Following digestion, the ventricles were minced and myocytes were suspended in Tyrode’s solution. For immunoblotting, myocytes were plated in laminin-coated polystyrene culture dishes and incubated in a solution of Medium-199 (catalog no. 31100-035; Gibco) containing Earle’s salts and l-glutamine at 37°C for 4 hr before treatment and collection.

*Immunoblotting*. We performed immunoblotting as described previously (Gao et al. 2013). Isolated ventricular myocytes were subjected to different treatments, including control and 5-min exposure to 10^–9^ M BPS, 10^–9^ M BPS + 10^–6^ M MPP, or 10^–9^ M BPS + 5 × 10^–6^ M PHTPP. Controls were treated with vehicle, and treatments were added to the culture medium. Treatments were stopped by aspirating the chemical-containing medium and washing cells with ice-cold phosphate-buffered saline (PBS). After centrifuging, the sedimented myocytes were snap frozen with liquid nitrogen. Equal amounts of protein extracts were separated by SDS-PAGE and transferred to a nitrocellulose membrane (Bio-Rad Laboratories). The membrane was blocked with 5% nonfat milk (supplemented with 0.1% Tween 20 in PBS) and incubated with primary antibodies and secondary antibodies. We used an enhanced chemiluminescence (ECL) Western blotting analysis system (GE Healthcare) for developing the membrane. We used antibodies against phosphorylated ryanodine receptor (pSer2808-RYR2 and pSer2814-RYR2), phosphorylated phospholamban (pSer16-PLN and pThr17-PLN), phospholamban (PLN-A1), and ryanodine receptor (RYR2), all from Badrilla; and horseradish peroxidase-conjugated anti-mouse and anti-rabbit secondary antibodies (Cell Signaling Technology). By using the phosphorylated protein-specific antibodies, we were able to detect the phosphorylated RyR and PLN at specific sites (i.e., pSer2808-RYR2, pSer2814-RYR2, pSer16-PLN, pThr17-PLN) as bands on the blots. The level of total RyR and PLN were detected using anti-RYR2 and anti-PLN-A1 antibodies, respectively. We compared the densities of bands from BPS, BPS + MPP, and BPS + PHTPP treatment with those of the control. Three female hearts and three male hearts were used for myocyte protein extractions.

*Myocyte mechanics, triggered activity analysis, and imaging of myocyte Ca^2+^ transients and sparks*. The acute effects of BPS or BPA on myocyte mechanics and Ca^2+^ handling were examined *in vitro* following rapid exposure of 1–7 min. Myocyte contraction and after contraction were recorded as previously described (Yan et al. 2011). Contractility was imaged with a charge-coupled device camera and a video-edge detector (Crescent Electronics). The myocytes were excited under field electrical pacing, which was generated by field stimulation with a Grass S48 stimulator (Grass Technologies). Myocytes were paced at a frequency of 0.5 Hz, with 2-msec pulses at an intensity of 1.5 times the threshold for activation. Data were sampled through an Axon Digidata 1322A board using the PCLAMP 9 software (both from Molecular Devices). After contraction was measured following electrical pacing at 2 Hz for 8 sec Ca^2+^ sparks were recorded from myocytes loaded with fluo-4 acetoxymethyl ester (5 μM; Molecular Probes) with a Zeiss LSM 710 inverted confocal microscope at excitation wavelength of 488 nm. Fluorescent signals were measured at > 515 nM in line-scan mode at 3.07-msec intervals, with each line comprising 512 pixels spaced at 0.056 mm. Ca^2+^ sparks are quantal, and spontaneous release of Ca^2+^ from the sarcoplasmic reticulum (SR) through the RyRs were observed as localized and transient rises in fluorescent intensity under confocal imaging. Analysis of Ca^2+^ spark images was performed using IDL 6.3 software (Exelis Visual Information Solutions). Ca^2+^ spark frequency was calculated as the number of sparks divided by the length of scan line, divided by the time of scan, and expressed as number of sparks per 100 μm per second. Ca^2+^ spark amplitude was calculated as the peak signal intensity of the spark and expressed as peak fluorescent intensity of the spark divided by baseline intensity (F/F_0_). To analyze Ca^2+^ transients, we measured fluorescence signals from myocytes loaded with fluo-4 acetoxymethyl ester using a Nikon TE 2000 microscope and an InCyt Standard PM photometry system (Intracellular Imaging). Data analysis and image processing were performed as previously described (Yan et al. 2011). A total of 17 female hearts and 7 male hearts were used as sources of isolated ventricular myocytes in these experiments.

*Statistical analysis*. All experiments were repeated independently using myocytes isolated from at least three rat hearts. We conducted one-way analysis of variance (ANOVA) with a multiple comparison post hoc test or *t*-test to compare differences between treatment groups. The chi-square test was used to analyze the frequency of events (e.g., the percentage of myocytes with triggered activities). We considered *p* < 0.05 to indicate statistical significance. Data were analyzed using SigmaPlot 11.0 and are expressed as mean ± SEM.

## Results

**Figure 2:**
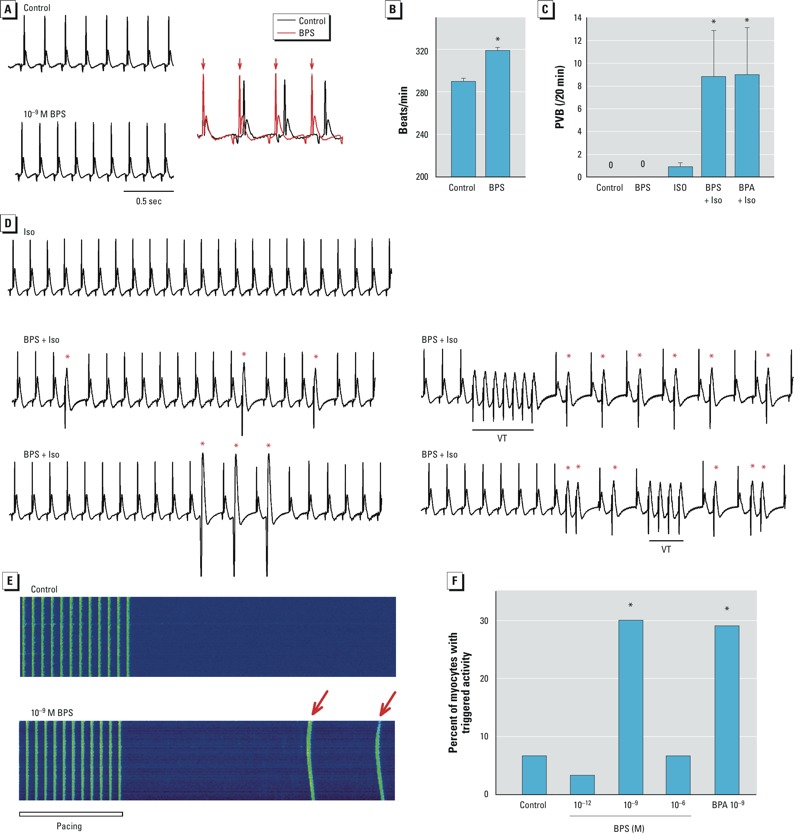
Effects of BPS exposure on female rat hearts and ventricular myocytes. (*A*) Left, surface ECG recording traces from female rat hearts under control conditions (vehicle) or in the presence of 10^–9^ M BPS. Right, overlay of the same traces showing increased heart rate in the presence of 10^–9^ M BPS. R-waves in in BPS-treated hearts are indicated by red arrows; the change in heart rate was observed within 5 min of BPS exposure. (*B*) Mean heart rate (± SEM) of female rat hearts under control conditions or in the presence of 10^–9^ M BPS (*n* = 3 hearts/group). (*C*) Mean frequency (± SEM) of premature ventricular beats (PVBs) in female rat hearts under control conditions or in the presence of 10^–9^ M BPS, 10^–8^ M Iso, BPS + Iso, or 10^–9^ M BPA + Iso (*n* = 6–8) hearts/group. (*D*) Representative surface ECG traces from female rat hearts treated with 10^–8^ M Iso alone or 10^–9^ M BPS + Iso; red asterisks indicate PVBs, and lines indicate ventricular tachycardia (VT); the two bottom traces represent BPS + Iso from the same heart. (*E*) Representative confocal images showing Ca^2+^ transients in female rat ventricular myocytes elicited by pacing under conditions or in the presence of 10^–9^ M BPS; red arrows indicate spontaneous Ca^2+^ transients (i.e., triggered activities) following electrical pacing. (*F*) Percentage of female rat ventricular myocytes having triggered activities; (*n* = 30 myocytes/group). *p < 0.05 compared with control by paired *t*-test (*B*), one-way ANOVA (*C*), or chi-square test (*F*).

*Effects of BPS on the electrical activities of* ex vivo *female rat hearts*. We examined the rapid effect of BPS on the electrical rhythm of *ex vivo* female rat hearts using surface ECG (Figure 2). All hearts showed normal sinus rhythms under control conditions. Exposure to 10^–9^ M BPS did not trigger detectable arrhythmia events (Figure 2A,C), but did result in a moderate increase in heart rate from 290 to 319 beats/min (*p* < 0.05; Figure 2A,B).

Under stress conditions created with the β-adrenergic agonist Iso (10^–8^ M), 10^–9^ M BPS markedly increased the frequency of premature ventricular beats (PVBs; Figure 2C,D) from 0.87 events/20 min with Iso alone, to 8.83 events/20 min with BPS + Iso (*p* < 0.05). Under stress conditions, the proarrhythmic effect of BPS was comparable to that of 10^–9^ M BPA; with BPA + Iso, the frequency of PVBs was 9.00 events/20 min (Figure 2C). In the presence of Iso, BPS also triggered episodes of nonsustained ventricular tachycardia (VT) in 1 of 6 female rat hearts (Figure 2D). We observed nonsustained VT in none of the hearts treated with Iso alone.

*Effects of BPS on triggered activities in female rat ventricular myocytes*. We examined the effect of BPS on the development of spontaneous excitations in female rat ventricular myocytes. These aberrant excitations, recorded as spontaneous Ca^2+^ transients after repeated pacing, are known as “triggered activities” (Bers 2002). Acute exposure to 10^–9^ M BPS for 2–7 min resulted in triggered activities in 30% of female rat ventricular myocytes (Figure 2E,F); by comparison, under control conditions, triggered activities were observed in 6.6% of myocytes (*p* < 0.05). The stimulatory effect of 10^–9^ M BPS on triggered activity was similar to that of 10^–9^ M BPA (Figure 2F). Unlike the robust effect seen at 10^–9^ M, 10^–12^ M or 10^–6^ M BPS did not alter the percentage of cells with triggered activities, giving rise to an inverted-U–shaped dose response (Figure 2F).

**Figure 3:**
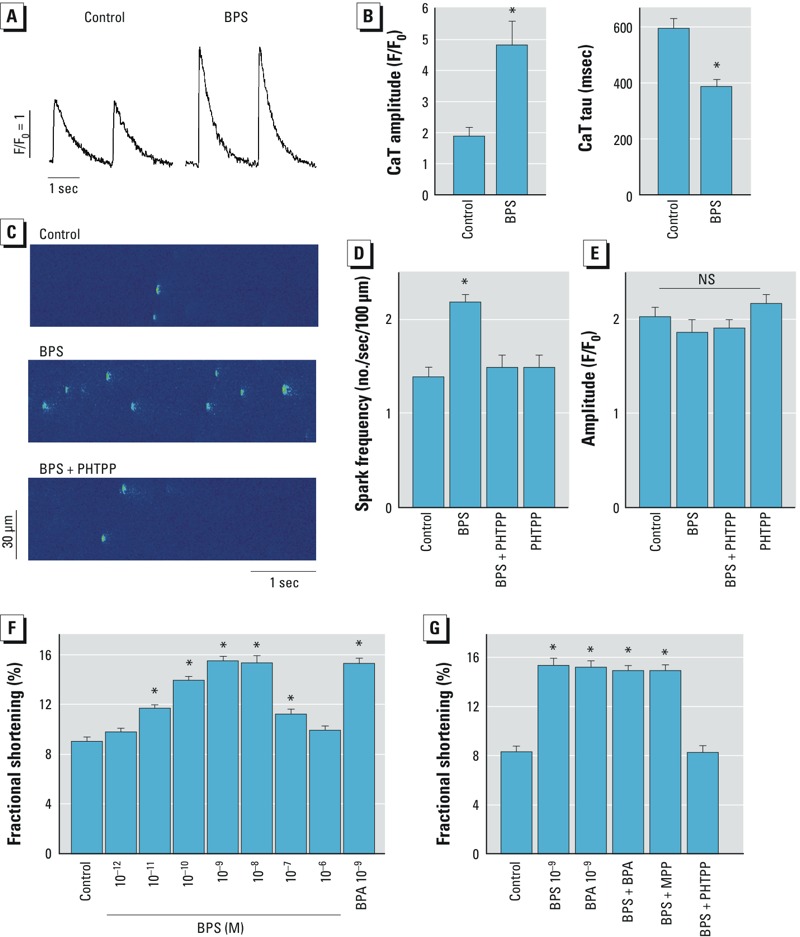
Effects of BPS on Ca^2+^ handling in female rat ventricular myocytes. (*A*) Ca^2+^ transient traces in female rat ventricular myocytes elicited by field electrical stimulation under control (vehicle) conditions or in the presence of 10^–9^ M BPS. (*B*) Mean (± SEM) Ca^2+^ transient (CaT) amplitude (left) and decay time constant (tau; right) under control conditions or in the presence of 10^–9^ M BPS (*n* = 6–8 myocytes/treatment). (*C*) Example confocal images of Ca^2+^ sparks in female rat ventricular myocytes under control conditions or in the presence of 10^–9^ M BPS or 10^–9^ M BPS + 5 × 10^–6^ M PHTPP. Mean (± SEM) Ca^2+^ spark frequency (*D*) and amplitude (*E*) in female rat ventricular myocytes under control conditions or in the presence of 10^–9^ M BPS, 10^–9^ M BPS + 5 × 10^–6^ M PHTPP, or PHTPP alone (*n* = 6–7 myocytes/treatment); NS, not significant. (*F*) Dose-dependent effect of BPS on the contractility (mean fractional shortening ± SEM) of female rat ventricular myocytes and the effect of 10^–9^ M BPA (*n* = 25–26 myocytes/treatment). (*G*) Mean (± SEM) fractional shortening of female rat ventricular myocytes under control conditions or in the presence of 10^–9^ M BPS, 10^–9^ M BPA, BPS + BPA, BPS + MPP (10^–6^ M), or BPS + PHTPP (5 × 10^–6^ M); *n* = 11–12 myocytes/treatment. *p < 0.05 compared with control by one-way ANOVA.

*Effects of BPS on Ca^2+^ handling in female rat ventricular myocytes*. Myocyte Ca^2+^ handling is fundamental to cardiac physiology; abnormal Ca^2+^ handling is a key mechanism of arrhythmogenesis (Bers 2002). We previously reported that alteration of myocyte Ca^2+^ handling plays an important role in the proarrhythmic action of BPA (Yan et al. 2011). Thus, we examined the acute effects of BPS (10^–9^ M) on Ca^2+^ handling in female rat ventricular myocytes. BPS rapidly increased the amplitude of field-stimulated Ca^2+^ transient (Figure 3A), from 1.88 (F/F_0_ ratio) under control conditions to 4.80 with BPS (Figure 3B). BPS also significantly decreased the time constant of field-stimulated Ca^2+^ transient (595.0 msec for control vs. 386.3 msec for BPS; Figure 3B), indicating an increase in SR Ca^2+^ reuptake. Increased diastolic SR Ca^2+^ release, or Ca^2+^ leak, plays a key role in the arrhythmogenic effect of BPA in the heart (Gao et al. 2013). SR Ca^2+^ release was assessed as Ca^2+^ sparks in quiescent female rat ventricular myocytes. Acute exposure to 10^–9^ M BPS significantly increased Ca^2+^ spark frequency, from 1.36 to 2.18 sparks/sec/100 μm (Figure 3C,D), without altering Ca^2+^ spark peak amplitude (Figure 3E) or temporal/spatial properties (data not shown). Blockage of estrogen receptor (ER) β with the selective blocker PHTPP completely abolished the effects of BPS on Ca^2+^ spark in female rat ventricular myocytes (Figure 3C,D). PHTPP alone in the absence of BPS had no detectable effect on Ca^2+^ sparks in female ventricular myocytes (Figure 3D,E).

Myocyte contraction, a manifestation of the Ca^2+^ cycling process, was used as a global index to further assess the effects of BPS. BPS enhanced fractional shortening of female ventricular myocytes in a dose-dependent manner, with an inverted-U–shaped dose–response curve (Figure 3F). The most efficacious effect of BPS (at 10^–9^ and 10^–8^ M) was comparable to the effect of 10^–9^ M BPA. The combination of BPS and BPA (10^–9^ M + 10^–9^ M) produce no increase in effectiveness, indicating no synergistic or antagonistic actions (Figure 3G). Selective blockage of ERβ with PHTPP, but not ERα with MPP, abolished the effects of BPS on myocyte contractility (Figure 3G).

**Figure 4:**
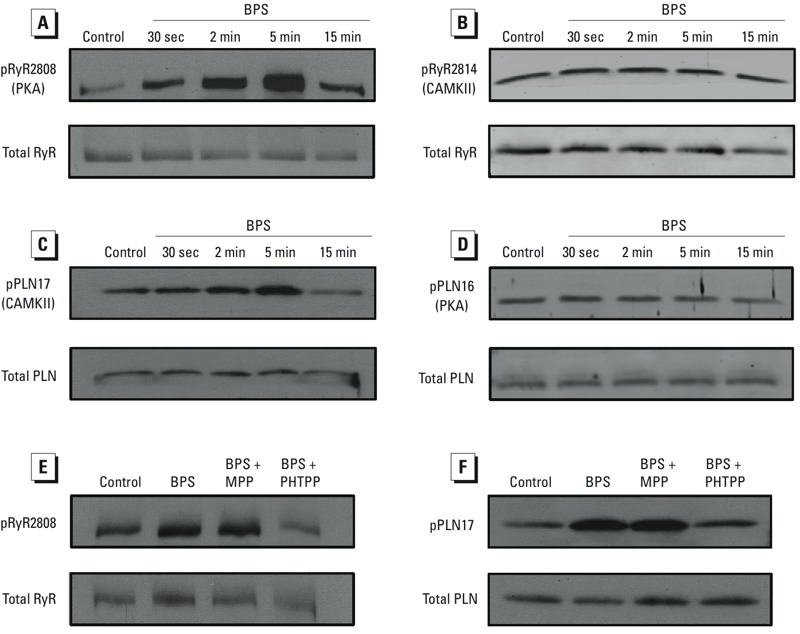
Effects of BPS on ryanodine receptor (RyR) and phospholamban (PLN) phosphorylation in female rat ventricular myocytes. Immunoblots of phosphorylated RyR at serine 2808 (PKA site; *A*) and serine 2814 (CAMKII site; *B*), along with total RyR from female rat ventricular myocytes under control conditions or in the presence of 10^–9^ M BPS for the indicated times. Immunoblots of phosphorylated PLN at threonine 17 (CAMKII site; *C*) and serine 16 (PKA site; *D*), along with total PLN from female rat ventricular myocytes under control conditions or in the presence of 10^–9^ M BPS for the indicated times. (*E*) Immunoblots of phosphorylated RyR at serine 2808 (PKA site) and total RyR from female rat ventricular myocytes under control conditions or in the presence of 10^–9^ M BPS, BPS + 10^–6^ M MPP, or BPS + 5 × 10^–6^ M PHTPP for 5 min. (*F*) Immunoblots of phosphorylated PLN at threonine 17 (CAMKII site) and total PLN from female rat ventricular myocytes under control conditions or after 5 min exposure to 10^–9^ M BPS, 10^–9^ M BPS + 10^–6^ M MPP, or 10^–9^ M BPS + 5 × 10^–6^ M PHTPP.

*Effects of BPS on phosphorylation status of Ca^2+^-handling proteins in female rat ventricular myocytes*. RyR and PLN are two key Ca^2+^-handling/regulatory proteins that can be phosphorylated to modify SR release and reuptake of Ca^2+^ (Kranias and Hajjar 2012; Van Petegem 2012). We examined the effects of BPS on the phosphorylation status of RyR and PLN by Western blot analysis of female rat ventricular myocytes (Figure 4). Similar to the effects of BPA (Gao et al. 2013), 10^–9^ M BPS rapidly and transiently increased phosphorylation of RyR at the protein kinase A (PKA) site serine 2808 (Figure 4A), and PLN at the Ca^2+^/calmodulin-dependent protein kinase II (CAMKII) site threonine 17 (Figure 4C). The effects of BPS on RyR and PLN phosphorylation were detectable after 30 sec of exposure and peaked at 5 min. BPS did not affect the phosphorylation of RyR at the CAMKII site (serine 2814; Figure 4B) or PLN at the PKA site (serine 16; Figure 4D). Selective blocking of ERβ with PHTPP, but not ERα with MPP, completely abolished the effects of BPS on the phosphorylation status of RyR as well as PLN (Figure 4E,F). PHTPP alone had no detectable effect on the phosphorylation of RyR or PLN (not shown).

**Figure 5:**
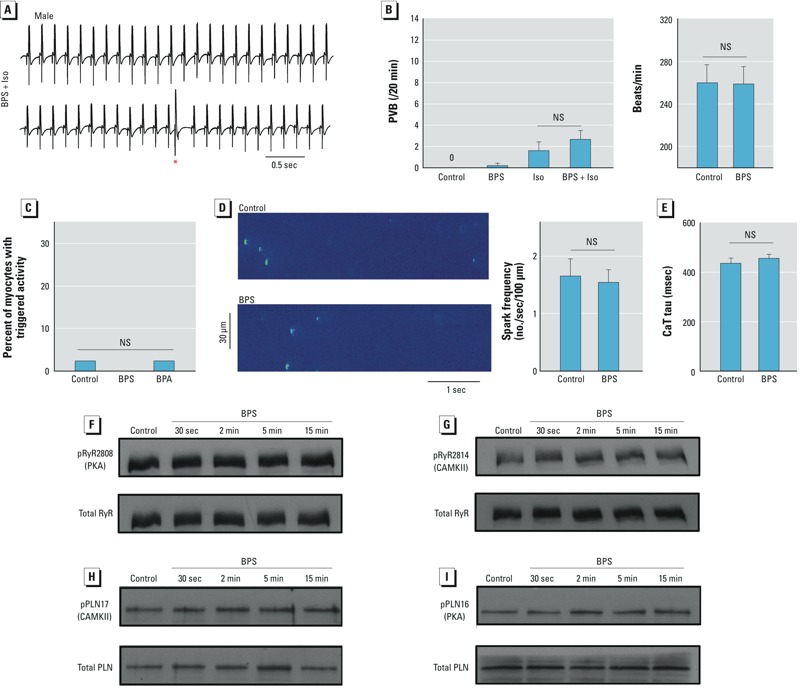
Effects of BPS on male rat hearts and ventricular myocytes. (*A*) Example surface ECG traces from male rat hearts treated with 10^–9^ M BPS + 10^–8^ M Iso; the red asterisk indicates premature ventricular beats (PVB). (*B*) Left, mean (± SEM) frequency of PVBs in male rat hearts under various conditions (*n* = 5–6 hearts/group). Right, mean (± SEM) heart rate (beats per min) of male rat hearts under control conditions and in the presence of 10^–9^ M BPS (*n* = 5 hearts/group). NS, not significant. (*C*) Percentage of male rat ventricular myocytes having triggered activities (*n* = 42–43 myocytes/group). (*D*) Left, example confocal images of Ca^2+^ sparks in male rat ventricular myocytes under control conditions and in the presence of 10^–9^ M BPS. Right, mean (± SEM) Ca^2+^ spark frequency under control conditions and in the presence of 10^–9^ M BPS (*n* = 10 myocytes/group). (*E*) Mean (± SEM) Ca^2+^ transient time constant (tau) under control conditions or in the presence of 10^–9^ M BPS (*n* = 13 myocytes/group). Immunoblots of phosphorylated RyR at serine 2808 (PKA site; *F*) and serine 2814 (CAMKII site; *G*), along with total RyR from male rat ventricular myocytes under control conditions and after exposure to 10^–9^ M BPS. Immunoblots of phosphorylated PLN at threonine 17 (CAMKII site; *H*) and serine 16 (PKA site; *I*), along with total PLN from male rat ventricular myocytes under control conditions and after exposure to 10^–9^ M BPS.

*Responses to BPS in male rat hearts and ventricular myocytes*. In striking contrast to the proarrhythmic effects observed in female heart, BPS had no detectable rapid action in male rat hearts. At the male whole-heart level, the frequency of ectopic beats after treatment with 10^–9^ M BPS + Iso was similar to that with Iso alone (*p* > 0.3), and 10^–9^ M BPS had no effect on the heart rate (Figure 5A,B). At the cellular level, BPS did not increase the incidence of triggered activities in male cardiac myocytes (Figure 5C) or alter the frequency of Ca^2+^ sparks (Figure 5D) or the kinetics of Ca^2+^ transient (Figure 5E). At the protein level, rapid exposure to BPS did not alter the phosphorylation levels of RyR by PKA or CAMKII (Figure 5F,G) or of PLN by PKA or CAMKII (Figure 5H,I).

**Figure 6:**
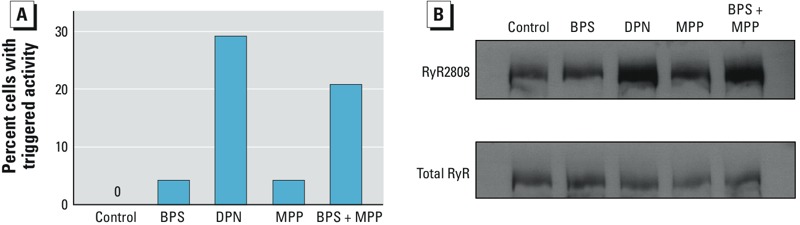
Percentage of male rat ventricular myocytes with triggered activities (*A*) and immunoblots of phosphorylated RyR at serine 2808 (PKA site) and total RyR from male rat ventricular myocytes (*B*) under control conditions or after treatment with 10^–9^ M BPS, 10^–7^ M DPN, 10^–6^ M MPP, or BPS + MPP.

Using incidence of triggered activity and PKA phosphorylation of RyR serine 2808 as end points, we examined the mechanism underlying the lack of response of male myocytes to BPS (Figure 6). Although BPS did not increase the incidence of triggered activities (Figure 6A) or RyR phosphorylation (Figure 6B), activation of ERβ with selective agonist DPN had marked stimulatory effects on these end points. Interestingly, the stimulatory effects of BPS were revealed under ERα blockage with MPP (MPP alone had no measurable effects) (Figure 6A,B).

## Discussion

With recognition of the potential adverse health impact of BPA exposure, BPS—a member of the bisphenol family with a structure similar to that of BPA—is increasingly used as a BPA alternative in the production of consumer goods; Liao and Kannan (2013) recently showed that BPS is becoming a common environmental chemical. Although there have been extensive studies of BPA, current knowledge on the biological and potential toxicological effects of BPS and other BPA substitutes is limited. In the present study, we observed that low-dose BPS had rapid impact in female rat hearts at the organ, cellular, and protein levels. These actions were female specific and are strikingly parallel to the proarrhythmic cardiac effects of an equal dose of BPA (Yan et al. 2011). These results indicate that BPS, at environmentally relevant doses, may also have potential adverse effects on female hearts and suggest that BPS (and possibly other BPA substitutes) is not necessarily free of the adverse health effects associated with BPA.

Limited, but growing, evidence suggests that BPS has potential estrogenic endocrine-disrupting activities. BPS, mostly at supraphysiological doses (micromolar to millimolar), has been reported to have genomic estrogenic activities in heterologous cell lines (Grignard et al. 2012; Hashimoto et al. 2001; Kuruto-Niwa et al. 2005). Recent studies found that BPS exposure (nanomolar dose range) had adverse effects on reproduction and progeny generation in zebrafish (Ji et al. 2013) and altered progesterone, testosterone, and cortisol synthesis in human adrenal cortico-carcinoma cells (Rosenmai et al. 2014). Viñas and Watson (2013) found that acute exposure to low-dose BPS activated ERK (extracellular signal-regulated kinase) rapid signaling in a pituitary cell line. In addition, Yamasaki et al. (2004) reported that exposure to BPS (20 or 500 mg/kg dose) for 3 consecutive days increased the uterine weight in female rats.

In the present study, we observed that at the myocyte level, a nanomolar concentration of BPS rapidly increased the incidence of aberrant spontaneous excitation in female myocytes. These “triggered activities” are single cell arrhythmia events that can propagate in the myocardium under certain pathophysiological conditions, and are well recognized as one of the central arrhythmogenic mechanisms in the heart (Bers 2002). At the whole-heart level, exposure to BPS alone resulted in a moderate increase in heart rate but did not trigger any arrhythmia events. We previously showed that BPA resulted in a rapid increase of intracellular cAMP in female rat ventricular myocytes (Gao et al. 2013). The pacemaker current—and, consequently, the rate of automaticity of cardiac nodal cells—are subject to regulation by cAMP, both directly and through PKA (Baruscotti and Difrancesco 2004). Although we did not investigate the effect of BPS on pacemaker cells in the present study, it is possible that a cAMP-mediated mechanism is responsible for the effect of BPS on heart rate. The effect of BPS on cardiac rhythm was also examined under catecholamine-induced stress condition. β-Adrenergic stimulation increases Ca^2+^ influx and SR Ca^2+^ load, and favors SR overload and abnormal Ca^2+^ release, thereby providing a substrate that favors arrhythmogenesis (Bers 2002). Under such stress conditions, BPS exposure triggered frequent premature ventricular beats, one of the most common forms of ventricular arrhythmias; in parallel comparisons, the effects of 10^–9^ M BPS on incidence of ectopic ventricular beats were indistinguishable from those of 10^–9^ M BPA. These results suggest that although BPS exposure by itself does not trigger clinically relevant arrhythmia events in healthy hearts under normal conditions, it may contribute to the development of arrhythmias in the presence of existing arrhythmogenic substrates such as elevated sympathetic tone and cardiac diseases.

The mechanism of action of BPS in female cardiac myocytes appears to be similar to that of BPA and involved rapid alteration of myocyte Ca^2+^ handling. BPS significantly and rapidly increased SR Ca^2+^ reuptake and, importantly, diastolic SR Ca^2+^ release, or Ca^2+^ leak, a pathophysiological change that plays a key role in arrhythmogenesis in cardiac diseases (Chelu and Wehrens 2007). In a previous study, we observed that suppression of SR Ca^2+^ leak blocked BPA-induced triggered activities in female cardiac myocytes (Yan et al. 2011). The effect of BPA on myocyte Ca^2+^ handling was mediated by characteristic impact on the phosphorylation of two key Ca^2+^ handling proteins, RyR and PLN (Gao et al. 2013). A nearly identical impact was also observed for BPS. RyR is the Ca^2+^ release channel of SR in cardiac myocytes, and its activity is subject to regulation by kinase phosphorylation. The two major phosphorylation sites are serine 2808 by PKA and serine 2814 by CAMKII. Either site’s phosphorylation by an individual kinase increases RyR opening probability via decreasing the threshold of Ca^2+^ sensor (Van Petegem 2012). PLN, on the other hand, regulates the Ca^2+^ reuptake process by its inhibitory interaction with sarco/endoplasmic reticulum Ca^2+^-ATPase (SERCA) (Kranias and Hajjar 2012). PLN also has two major phosphorylation sites, serine 16 (PKA) and threonine 17 (CAMKII). Phosphorylation of either site releases PLN’s inhibition of SERCA, thereby increasing Ca^2+^ reuptake into the SR (Kranias and Hajjar 2012). In the present study, we observed that BPS exposure rapidly and transiently increased phosphorylation of RyR by PKA but not by CAMKII, and PLN by CAMKII but not PKA. The time course for BPS and the target-specific manner of BPS’s effect are remarkably similar to those described for the cardiac actions of BPA (Gao et al. 2013). Upstream, we observed that the impact of BPA on RyR and SR Ca^2+^ release is mediated by the adenylyl cyclase-cAMP-PKA signaling pathway, and the impact on PLN is mediated by the phospholipase C-inositol trisphosphate receptor-CAMKII pathway (Gao et al. 2013). A similar upstream signaling mechanism may also mediate the rapid actions of BPS.

With respect to the pharmacodynamics of BPS action, in heterologous expression systems BPS had potency (median effective concentration) similar to that of BPA in activating an estrogen-responsive GFP (green fluorescent protein) reporter gene (Kuruto-Niwa et al. 2005); yet, in a more recent study, Molina-Molina et al. (2013) reported that BPS was > 20-fold less potent than BPA in activating an estrogen-responsive luciferase reporter gene, was less potent in activating ERα and ERβ-driven luciferase activities, and had lower affinity in binding to ERα and ERβ in a competitive binding assay. In addition, Kitamura et al. (2005) found BPS to have antiandrogenic effects with a potency lower than that of BPA. In the present srudy, we found that the pharmacodynamic properties of BPS’s rapid action in cardiac myocytes closely resembled those of BPA. Using incidence of arrhythmogenic triggered activity and myocyte mechanics as end points, the dose–response curve of the rapid effects of BPS had a nonmonotonic shape, similar to that described for BPA (Liang et al. 2014). The two chemicals have similar potency and efficacy, and the same most efficacious dose around 10^–9^ to 10^–8^ M. These findings contrast with the lower potency of BPS compared with BPA reported in other studies (Kitamura et al. 2005; Molina-Molina et al. 2013). Nonmonotonic dose responses are commonly observed for hormones and EDCs and have important implications for the assessment of toxicity of EDCs (Vandenberg et al. 2012b). Previously, we demonstrated that the nonmonotonic dose response of BPA is the result of multiple monotonic actions on individual elements of myocyte Ca^2+^ handling processes, and the combined effect of its stimulatory effect on SR Ca^2+^ release and reuptake and the opposing inhibitory effect on Ca^2+^ influx through the L-type Ca^2+^ channel (Liang et al. 2014). These findings may provide clues to the mechanism giving rise to the nonmonotonic dose response of BPS in the heart.

There is striking contrast in the responses of female and male rat hearts to rapid low-dose BPS exposure. Although female hearts responded robustly to BPS at the organ, cellular, and protein levels, no detectable responses were found in male hearts using those same end points. Importantly, BPS produced no proarrhythmic effects, including increased ectopic beats or ventricular tachycardia, in male hearts under Iso-induced stress, nor did it increase the incidence of arrhythmic activities in isolated male myocytes. At the cellular and protein levels, BPS exposure rapidly altered myocyte SR Ca^2+^ handling and phosphorylation status of key Ca^2+^-handling proteins in female hearts; these effects were absent in male rat hearts. In previous studies, we demonstrated that such remarkable sex-specific cardiac response to estrogenic chemicals is due to the opposing and counterbalancing actions of ER signaling (Belcher et al. 2012). We observed that in both male and female rat cardiac myocytes, ERβ signaling had stimulatory effects on Ca^2+^ handling and development of triggered activity, but ERα signaling was inhibitory. The response of the heart was determined by the balance of ERβ and ERα signaling. Thus, the proarrhythmic effect of estrogenic chemicals in female rodent hearts was dominated by the stimulatory ERβ rapid signaling, whereas in male hearts the effect of ERβ was present but masked by the inhibitory ERα signaling, resulting in a lack of observable response. Results shown in Figure 6 suggest that a similar mechanism mediates the lack of response of male cardiac myocytes to BPS. Consistent with this model, activation of ERβ alone by DPN revealed that the stimulatory effect of ERβ signaling was intact in male cells; in the presence of ERα blockage, the stimulatory effect of BPS (presumably via ERβ) was observed.

## Conclusions

We observed that rapid exposure to low-dose BPS had proarrhythmic impact on female rat hearts through mechanisms involving activation of ERβ signaling and alteration of myocyte Ca^2+^ handling. The cardiac actions of BPS, as measured by multiple end points, are similar to those previously reported for BPA; also similar are the cellular and molecular mechanisms underlying the effects of the two chemicals. As a case study, our findings suggest that BPS and other structurally related BPA substitutes may share similar endocrine-disrupting activities with BPA.
